# Inhibition of Hyperpolarization-Activated Cation Current in Medium-Sized DRG Neurons Contributed to the Antiallodynic Effect of Methylcobalamin in the Rat of a Chronic Compression of the DRG

**DOI:** 10.1155/2015/197392

**Published:** 2015-05-26

**Authors:** Ming Zhang, Wenjuan Han, Jianyong Zheng, Fancheng Meng, Xiying Jiao, Sanjue Hu, Hui Xu

**Affiliations:** ^1^Institute of Neurosciences, the Fourth Military Medical University, Xi'an 710032, China; ^2^State Key Laboratory of Cancer Biology and Institute of Digestive Diseases, Department of Digestive Surgery, Xijing Hospital, the Fourth Military Medical University, Xi'an 710032, China

## Abstract

Recently several lines of evidence demonstrated that methylcobalamin (MeCbl) might have potential analgesic effect in experimental and clinical studies. However, it was reported that MeCbl had no effect on treating lumbar spinal stenosis induced pain. Thus, the effects of short-term and long-term administration of MeCbl were examined in the chronic compression of dorsal root ganglion (CCD) model. We found that mechanical allodynia was significantly inhibited by a continuous application of high dose and a single treatment of a super high dose of MeCbl. Little is known about mechanisms underlying the analgesia of MeCbl. We examined the effect of MeCbl on the spontaneous activity (SA), the excitability, and hyperpolarization-activated nonselective cation ion current in compressed medium-sized dorsal root ganglion (DRG) neurons using extracellular single fiber recording *in vivo* and whole-cell patch clamp *in vitro*. We found that MeCbl significantly inhibited the SA of A-type sensory neurons in a dose-dependent manner and inhibited the excitability of medium-sized DRG neurons. In addition, MeCbl also decreased *I*_*h*_ current density in injured medium-sized DRG neurons. Our results proved that MeCbl might exert an analgesic effect through the inhibition *I*_*h*_ current and then might inhibit the hyperexcitability of primary sensory neurons under neuropathic pain state.

## 1. Introduction

Methylcobalamin (MeCbl) has a strong affinity for nerve tissues [1, 2]. Furthermore, MeCbl participates in DNA and protein methylation as a coenzyme of methionine synthase in the transmethylating action [3–5]. MeCbl plays an important role in myelination, neuronal differentiation and replication, and cellular activity in nerve tissues [6, 7]. Combined with other medicines, MeCbl has always been used to treat B12 deficiency and Alzheimer's disease syndromes [8, 9]. Evidence showed that the coapplication of MeCbl and pioglitazone instinctively decreased allodynia and hyperalgesia in diabetic rats [10], and the combination of MeCbl and vitamin E alleviated thermal hyperalgesia in sciatic nerve crush injured rats [11]. Very few studies reported that MeCbl treatment relieved paresthesia, burning pain, and spontaneous pain in neuropathic patients [12, 13] and MeCbl alleviated subacute herpetic, trigeminal, and glossopharyngeal neuralgia [14–16]. Several clinical trials confirmed the efficacy and safety of MeCbl in relieving pain and improving axonal degeneration and nerve conduction velocities for diabetic peripheral neuropathy [17, 18]. But there was evidence that MeCbl lacked effectiveness in treating lumbar spinal stenosis induced pain [19]. In addition, the precise mechanism of MeCbl's effect on peripheral neuropathy remains obscure. In the present study, we evaluated the effect of monotherapy of MeCbl in the chronic compression of dorsal root ganglion (CCD) model, which mimics low back pain and lumbar spinal stenosis syndromes well.

Mounting evidence suggests that a possible cause of low back pain and radicular pain is the mechanical deformation of the dorsal root ganglion (DRG) and its nerve roots, resulting from spinal stenosis, radiculopathies, and tumors [20]. The animal model of CCD mimics low back pain and radicular pain syndromes in the rat [21]. Previous studies showed that DRG neurons exhibited hyperexcitability, including spontaneous activity, increased excitability, decreased potassium current, and upregulation of the hyperpolarization-activated cation current (*I*_*h*_) in the CCD rat [22–25].

The hyperexcitability of DRG neurons associated with an injury of a peripheral nerve or the sensory ganglion may contribute to pain-related behaviors in experimental animal models of neuropathic pain. *I*_*h*_ regulates neuronal excitability by limiting membrane hyperpolarization and facilitating depolarization [24, 26]. *I*_*h*_ exists widely in primary sensory neurons [27, 28]. *I*_*h*_ blocker ZD7288 inhibited the SA originated from injured DRG neurons in animal models of SNL and CCI rats [29–31]. Many medium-sized sensory neurons are responsive to thermal and/or mechanical noxious stimuli through peripheral nociceptors. Others transmit nonnociceptive information as low-threshold mechanoreceptors [32]. The low-threshold type of medium-sized DRG neurons might not be normally involved in acute pain but might contribute to tactile allodynia in pathological cases. Thus, the effect of MeCbl on the SA, the excitability, and *I*_*h*_ in medium-sized DRG neurons were further determined in CCD rats in the present study.

## 2. Materials and Methods

### 2.1. Animals and Surgery

Adult female Sprague-Dawley (SD, 120 g–250 g, *n* = 132) rats were used in experiments, according to the guildlines for the care and use of laboratory animals and the protection committee of our university. The CCD model was prepared according to the previous method [21]. The sterilized surgical procedures were carried out under sodium pentobarbital anesthesia (40 mg/kg, i.p.). The skin was incised on the left side of the lumbar vertebrae between L4 and L6 and the left paraspinal muscles were separated from the mammillary process and the transverse process at the L4–L6 level. In the first group of rats (the CCD group), the L5 intervertebral foramen was clearly exposed and a fine, L-shaped needle inserted about 4 mm into the L5 intervertebral foramen at a 30° angle with respect to the dorsal middle line and 10° with respect to the vertebral horizontal line. When the needle tip reached the DRG, the hind leg muscles of the operated side exhibited a slight, transient twitch. Then, the needle was withdrawn from the L5 intervertebral foramen and a stainless steel rod, 4 mm in length and with different diameters, was inserted into the L5 intervertebral foramen along the path of the needle. The diameter of the stainless steel rod was increased in relation to the weight of the rat; for example, a diameter of 0.4 mm was selected for the rats of 120–150 g and a diameter of 0.5 mm and 0.6 mm for the rats of 151–200 g and 201–250 g, respectively [21]. Then the muscular layer and skin were sutured. To isolate recording and administrating pools for the experiments of single-fiber recording, L5 DRG was compressed in the rat. To obtain more intact DRG samples for the whole-cell patch clamp recording, both L4 and L5 DRGs were compressed in the rat. As for the sham group, the surgical procedure was identical to that for the CCD group, except that the L-shaped needle was not inserted into the intervertebral foramen.

### 2.2. Pain Behavioral Test of Mechanical Allodynia

Fifty-two SD rats were housed in a plastic cage (40 cm × 60 cm × 30 cm) with soft bedding and free access to food and water and a 12 h day/12 h night cycle. These animals were divided randomly into CCD and sham groups.

Rats were placed in plastic cage with metal mesh floor and allowed to habituate for at least 30 min before testing. Mechanical stimuli, produced by Von Frey filaments (Stoelting Co., USA), were used to assess the bilateral paw withdrawal threshold. Every rat was placed on a metal mesh floor in a plastic cage (20 cm × 25 cm × 15 cm). A series of von Frey filaments (1.0–15.0 g) touched the plantar part of the hindpaw in the ascending order [33]. Flicking, withdrawal, or licking of the injured hindpaw was treated as positive response to nociception. Each filament was applied 5 times to its bending force, and a paw withdrawal threshold was defined as the minimal force of filaments inducing three or more positive responses. In order to prevent the tissue from injury, the cut-off threshold was assigned at 15.0 g. Dosage of MeCbl injection solution (Eisai, Japan) was administrated according to rat's weight. To study the short-term effect of MeCbl on neuropathic pain, MeCbl was intraperitoneally injected to CCD rats on the 3rd postoperative day for one time. MeCbl (1.25, 2.5, and 10 mg/kg) and vehicle (2.5 and 10 mg/kg) were, respectively, injected in CCD and sham rats. MeCbl was also intraperitoneally and successively injected every day from the 3rd postoperative day.

Mannitol (Chemscene, Japan) is the supplementary material in the MeCbl injection. The maximal concentration of Mannitol was considered as vehicle in the experiment.

### 2.3. Electrophysiological Recording

#### 2.3.1. Extracellular Recording of A-Type Single-Fiber Activities of L5 Dorsal Root* In Vivo*

The spontaneous activity of DRG single fibers was obtained from CCD rats. All animals at postoperative 3–7 days were used for extracellular recording of single fiber* in vivo*. Rats were anesthetized with mixture of anesthetics (*α*-chloralose 20 mg/kg and urethane 340 mg/kg, i.p.). The laminectomy was performed at L1-L2 and L4-L5 and a small pool was prepared at each of the two sites. In the L4-L5 pool, the stainless steel rod was taken out and the compressed L5 DRG was sufficiently exposed. The spinal nerve was transected about 5–10 mm distal to the ipsilateral L5 DRG so that the discharges were recorded from L5 dorsal root fibers originated primarily from the L5 ganglion and not from receptors in peripheral tissue. During the experiment, the L4-L5 pool was filled with 1 mL of warm Kreb's solution (35–37°C) containing (in mM) NaCl 150, KCl 5, CaCl2 2, MgCl2 1, D-glucose 10, and HEPES 10, with the pH adjusted to 7.4. In the L1-L2 pool, the dorsal root of the L5 DRG was covered with warm paraffin oil (35–37°C). Point thermometer (TM-1320, OBEST) was applied to detect temperature of two pools during experiment. And the rats lay on the linear animal temperature regulator (BME-461A, CAMS) which was kept at 37°C. Under the microscope, a bundle of L5 dorsal root (about 10 *μ*m in diameter) which might include one microfilament or a few nerve fibers was isolated and cut off. Then the broken end of fractured bundle was located to a fine platinum electrode (29 *μ*m in diameter) for electrophysiological recording. The firing patterns of A-type fiber bundle were displayed in a memory oscilloscope (VC-11, Japan) and collected via an A/D board to a computer hard drive and stored for offline analysis. Unitary firings were identified according to their spike waveforms, and those microfilaments with single-fiber firings were used for further recording [21, 34].

The spontaneous activity of A-type dorsal root was recorded for no less than 3 min, and then MeCbl or vehicle was administrated to L5 DRG, respectively. The mean firing rate of about 3 min before application of MeCbl was used as the basal frequency of SA. The inhibitory rate of MeCbl or vehicle was calculated by the formula: inhibitory rate = (basal frequency of SA in the absence of MeCbl or vehicle − the frequency of SA in the presence of MeCbl or vehicle)/basal frequency of SA × 100%. The percentage changes of firing rates were considered significant if the changes were 15% or greater [34].

#### 2.3.2. Whole-Cell Patch Clamp Recording of Medium-Sized DRG Neurons* In Vitro*

CCD rats of postoperative 3−7 days and sham rats were anesthetized with pentobarbital sodium (40 mg/kg, i.p.). L4 and L5 DRGs on the left were carefully exposed and the spinal nerve connected to DRG was reserved as long as possible (>2 cm) in the preparation. Intact L4 and L5 DRGs and attached spinal nerve were carefully removed from the vertebral column and placed into artificial cerebrospinal fluid (ACSF (in mM): NaCl 124, KCl 2.5, NaH_2_PO_4_ 1.2, MgCl_2_ 1.0, CaCl_2_ 2, NaHCO_3_ 25, and Glucose 10). Spinal dura maters and connective tissues nearby were cleaned, and then the intact ganglia were digested in a digestive solution (2 mg/mL trypsin and 3.2 mg/mL type-A collagenase) for 45 min at 37°C and were agitated by gentle bubbling with mixed gas (95% O_2_ and 5% CO_2_). The intact ganglia were transferred into ACSF and incubated in ACSF with mixed gas at 28°C for at least 1 hour before being moved into the recording chamber.

During the recording, the chamber was filled with different solutions. These bath solutions (ACSF) were saturated with 95% O_2_ and 5% CO_2_. The end of the spinal nerve was sucked by a stimulating electrode. And the conduction velocity (CV) of medium-sized DRG soma was measured as the distance between the stimulating electrode and the recording electrode divided by the time between the electrical stimulus and the evoked AP. Individual neurons could be visualized with a 40 × water-immersion objective under a microscope (BX51WI; Olympus, Tokyo, Japan). Whole-cell current and voltage clamp recording were carried out by a multiclamp 700A amplifier (Molecular Devices Corporation, Sunnyvale, CA, USA). Patch pipettes (2–4 MΩ) were pulled from borosilicate glass capillaries and pulled on a micropipette puller (P-97, Sutter Instrument, USA). Neurons, in which membrane potentials were more negative than −50 mV and which exhibited overshooting action potentials, were selected for further study. Signals were digitized at 10 kHz by a 1320 A/D board (Molecular Devices, USA) and used for data acquisition and analysis. A gigaohm seal was reached by a small negative pressure, and the whole-cell recording was established by further negative pressure or a buzz signal. The membrane potential was held at −60 mV in the voltage clamp. And the data were discarded if the Ra or membrane potential changed by 20% during an experiment.

The DRG soma was classified visually by the diameter of its soma as small (<30 *μ*m), medium-sized (31–45 *μ*m), or large (>45 *μ*m) [22]. Medium-sized DRG somas were selected in our experiment. Passive and active membrane features were collected. Resting membrane potential, Cm (membrane capacitance), and Rm (membrane resistance) were recorded on line. Positive membrane features including AP amplitude, AP half-width, max-rise slope, and AHP amplitude were analyzed on the first evoked AP. The first action potential was evoked by the step wave from −100 pA to 1000 pA with an increase of 50 pA. AP amplitude (mV) was regarded as voltage difference between the resting membrane potential (RMP) and the peak upward. AP half-width was characterized as duration of AP after 50% repolarization. Max-rise slope was measured as differential on AP; the value of the peak point and AHP amplitude (mV) was voltage difference between the lowest point of hyperpolarization and the RMP.

In our experiment, the internal solution contained (in mM) K-gluconate 120, KCl 18, CaCl_2_ 1, MgCl_2_ 2, EGTA 5, HEPES 10, Na_2_-ATP, and Na_3_-GTP. Osmolarity was adjusted to 280–290 mOsm (pH = 7.4). The hyperpolarization potentials were got from −110 mV to −60 mV with an increment of 10 mV. In order to evaluate changes of *I*_*h*_ between CCD and sham rats, *I*_*h*_ current density was calculated as *I*_*h*_ current amplitude divided by Cm. MeCbl was perfused to intact DRGs for 5 min, and then the indices above were measured.

### 2.4. Statistics

All data were stored in computer hard drives for offline analysis and were expressed as mean ± sem. The P-clamp 9 software (Molecular Devices, USA) was used for whole-cell patch clamp data acquisition and analysis. Statistical evaluations were performed by using Statistical Product and Service Solutions (SPSS) software. The data were analyzed and painted by the software of sigmaplot (10.0), origin (8.0), and Coreldraw (X3). The change of SA, *I*_*h*_ current density, and passive and active membrane properties of medium-sized DRG neurons before and after the treatment of MeCbl were analyzed by Student's *t*-test (paired sample *t*-test). The number of APs, *I*_*h*_ current density, and passive and active membrane properties were compared by one-way analysis of variance (one-way ANOVA) between CCD and sham groups. One-way ANOVA was also used to test significant difference on inhibition rate at different concentration of MeCbl. Repeated-measures ANOVA with Bonferroni's post hoc test were used to compare the number of APs and mechanical behavior before and after the application of MeCbl. The pairwise comparison in the number of APs and mechanical behavior between CCD and sham groups were analyzed using multivariate analysis of variance (MANOVA). *P* < 0.05 was assumed to indicate statistical significance.

## 3. Results

### 3.1. Effects of MeCbl on Mechanical Allodynia

The mechanical paw withdrawal thresholds (PWTs) of CCD (*n* = 6) and sham rats (*n* = 6) were recorded for 21 days. After the surgery, both ipsilateral and contralateral PWTs of CCD rats (*n* = 6) were much decreased compared to those of sham rats during postoperative 21 days (*Ps* < 0.001, repeated-measures ANOVA). MeCbl was intraperitoneally injected during postoperative 3 to 10 days, and short-term and long-term applications of MeCbl on mechanical allodynia were investigated.

#### 3.1.1. A Short-Term Treatment of MeCbl Alleviated Mechanical Allodynia

**Figure 1:**
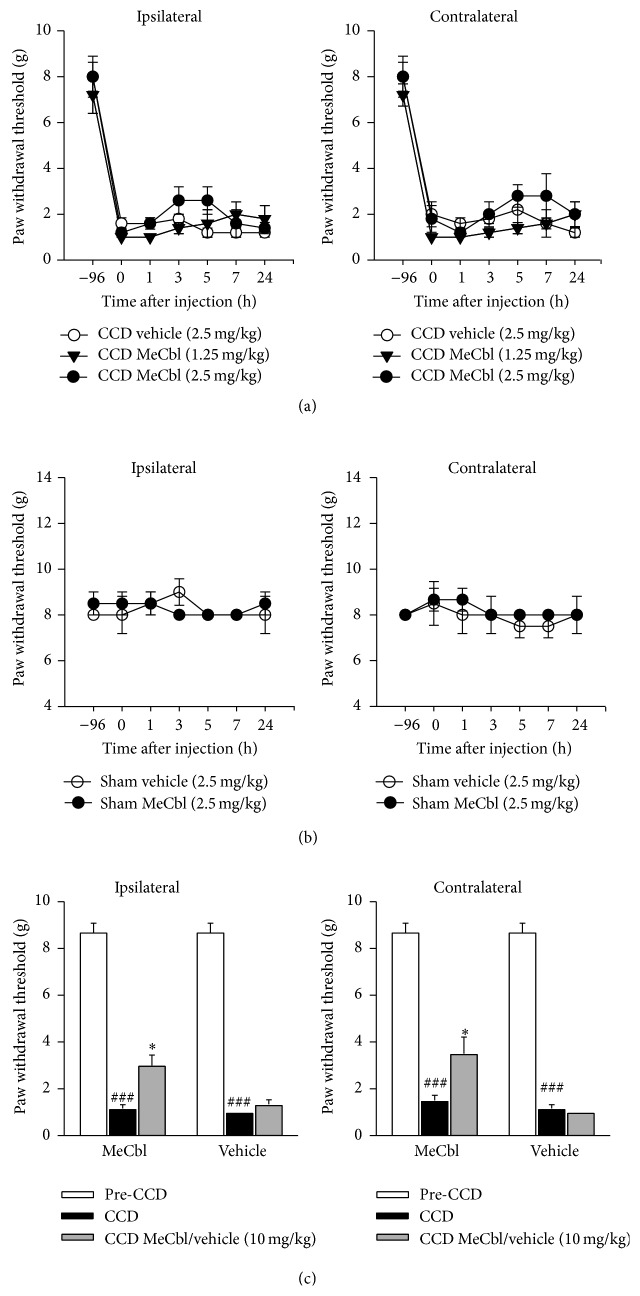
High dose of MeCbl ameliorated mechanical allodynia of rats following the CCD in the short term. MeCbl (1.25 mg/kg, 2.5 mg/kg, and 10 mg/kg) was injected intraperitoneally on the postoperative 3 to 10 days. (a) Effects of MeCbl on ipsilateral and contralateral mechanical paw withdrawal thresholds in CCD rats following the injection. (b) No effects of MeCbl on ipsilateral and contralateral mechanical paw withdrawal thresholds in sham rats. (c) A higher concentration of MeCbl affected bilateral paw withdrawal thresholds. ^###^ *P* < 0.001, compared to those in pre-CCD group; ^∗^ *P* < 0.05, compared with those of CCD group.

Following the single intraperitoneal injection of MeCbl, pain behavioral tests were examined in 1 h, 3 h, 5 h, 7 h, and 24 h. There were no significant differences in ipsilateral and contralateral PWTs of CCD rats after the short-term application of MeCbl (1.25 and 2.5 mg/kg) and vehicle (2.5 mg/kg) (*Ps* > 0.05, repeated-measures ANOVA, *n* = 5, Figure 1(a)). Moreover, MeCbl (2.5 mg/kg) did not affect bilateral PWTs of sham group compared to those of vehicle (*Ps* > 0.05, repeated-measures ANOVA, Figure 1(b)). The single treatment of a higher dose of MeCbl (10 mg/kg) was tested in 20 min to 30 min of the injection. Both ipsilateral and contralateral PWTs were enhanced in CCD rats following the injection of MeCbl (*Ps* < 0.05, paired *t*-test, Figure 1(c)). But vehicle did not significantly affect bilateral PWTs (*Ps* > 0.05, paired *t*-test, Figure 1(c)).

#### 3.1.2. A Continuous Treatment of MeCbl Ameliorated Mechanical Allodynia

**Figure 2:**
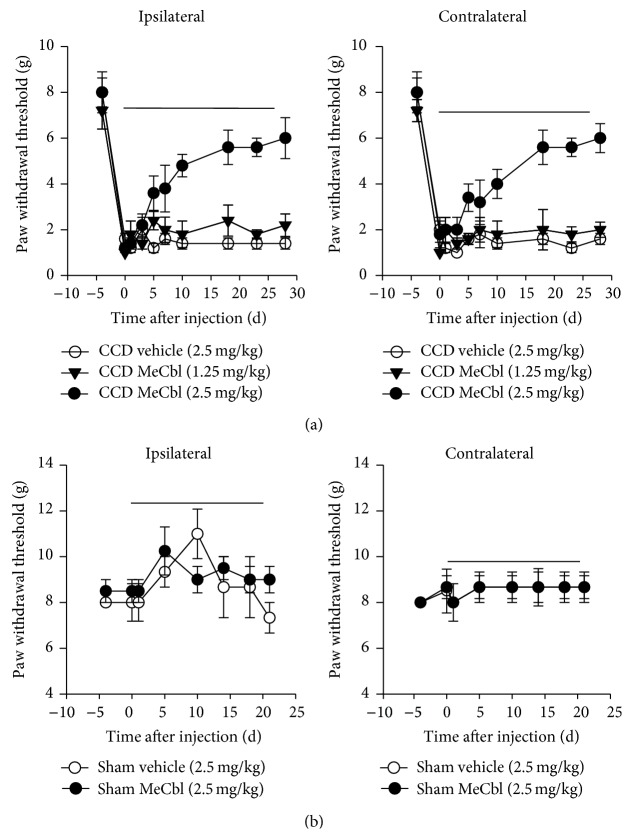
Continuous treatment of MeCbl inhibited bilateral mechanical allodynia of CCD rats. Continuous treatment of MeCbl (1.25 mg/kg and 2.5 mg/kg) or Mannitol (2.5 mg/kg) was injected intraperitoneally from the 3rd postoperative day (black line segment). (a) Effects of MeCbl on bilateral mechanical paw withdrawal thresholds of CCD rats. (b) Effects of MeCbl on mechanical paw withdrawal thresholds of sham rats.

MeCbl (1.25 mg/kg) was intraperitoneally injected continuously and once daily for 28 days, and there were no significant differences in bilateral PWTs between CCD and sham groups (*Ps* > 0.05, repeated-measures ANOVA, *n* = 5, Figure 2(a)), while MeCbl (2.5 mg/kg) significantly increased bilateral PWTs compared to vehicle (*Ps* < 0.001, repeated-measures ANOVA, *n* = 5, Figure 2(a)). Since the successive injection of MeCbl (2.5 mg/kg) for 5 days, bilateral PWTs were distinctly increased, compared to those of the vehicle group (*Ps* < 0.01, repeated-measures ANOVA, *n* = 5, Figure 2(a)).

Effects of MeCbl (2.5 mg/kg) and vehicle (Mannitol 2.5 mg/kg) were also studied on sham rats. MeCbl and Mannitol did not significantly affect bilateral PWTs of sham rats compared to vehicle group (*Ps* > 0.05, repeated-measures ANOVA, Figure 2(b)).

### 3.2. Inhibitory Effects of MeCbl on the SA of A-Type DRG Neurons

#### 3.2.1. MeCbl Inhibited the SA of A-Type DRG Neurons* In Vivo*

**Figure 3:**
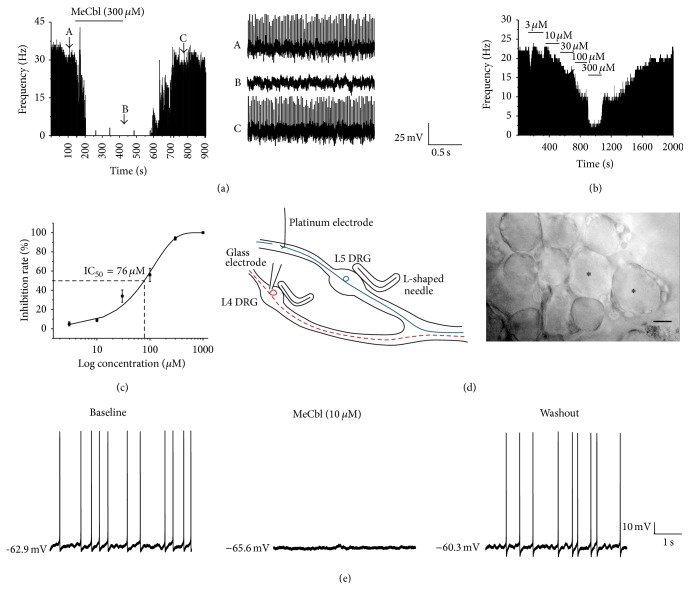
MeCbl inhibited spontaneous activity (SA) of A-type neurons of the compressed DRG in a dose-dependent way. (a) Time histogram shows that the application of MeCbl (300 *μ*M) decreases the basal firing rate of an A-type dorsal root fiber* in vivo*. Three traces in right panel were showed firing patterns before (A), during (B) and wash (C) the application of MeCbl. (b) Time histogram shows the inhibitory effect of MeCbl in a dose-dependent manner. (c) The concentration-response curve shows the value for IC_50_ (76 *μ*M) of MeCbl. (d) Schematic graph of whole-cell recording of DRG neurons and photography of medium-sized DRG neurons. Scale = 20 *μ*m. (e) MeCbl (10 *μ*M) inhibited the SA of one medium-sized DRG neuron of CCD group.

Among 62 units of microfilament of dorsal root exhibited stabled SA in CCD rats (*n* = 42, CCD rats), 66.1% (41/62) of those units were sensitive to MeCbl. The insensitive effect was considered as the percentage changes of firing rates lower than 15% in the presence of the maximal dose of MeCbl (300 *μ*M). Eighty-two point nine percent (34/41) could be recovered in 10 min after the washout (Figure 3(a)). MeCbl inhibited the SA in a dose-dependent manner (*n* = 5) from 3 to 300 *μ*M in CCD rats (*Ps* < 0.05, Student's *t*-test, Figure 3(b)). The 50% inhibition concentration of MeCbl was 76 *μ*M (Figure 3(c)).

#### 3.2.2. MeCbl Inhibited the SA of Medium-Sized DRG Neurons* In Vitro*

For A-type DRG neurons were recorded in the* in vitro* experiment; the medium-sized DRG neurons were selected from CCD and sham rats (CCD group: 37.7 ± 0.62 *μ*m, *n* = 36; sham group: 36.05 ± 0.57 *μ*m, *n* = 23). The conduction velocities of medium-sized DRG neurons were measured (11.20 ± 2.94 m/s, *n* = 7), which belonged to A*δ* DRG neurons. Among the 7 A*δ* neurons, 4 A*δ* neurons belong to CCD group and 3 A*δ* neurons come from sham group. Twenty-two point two (8/36) medium-sized neurons from the intact DRGs developed SA* in vitro*. After the application of MeCbl (10 *μ*M), the SA was eliminated during 60 s and the SA was recovered in 10 min after washout. About 50% of MeCbl-responsive neurons were reversible in medium-sized DRG neurons of CCD rats after washout (Figure 3(d)).

### 3.3. Inhibitory Effects of MeCbl on the Excitability of Medium-Sized DRG Neurons

#### 3.3.1. MeCbl Inhibited the Excitability of Medium-Sized DRG Neurons

The excitability of medium-sized DRG neurons was recorded in intact DRGs* in vitro* (*P* > 0.05, one-way ANOVA, CCD group: 38.91 ± 1.09 *μ*m, *n* = 17; sham group: 35.51 ± 0.54 *μ*m, *n* = 15). No significant differences were found in passive membrane properties between the sham and CCD groups (*Ps* > 0.05, *n* = 32, Student's *t*-test, Table 1). And MeCbl did not affect passive membrane properties in sham rats either (*Ps* > 0.05, *n* = 15, Student's *t*-test, Table 1).

**Figure 4:**
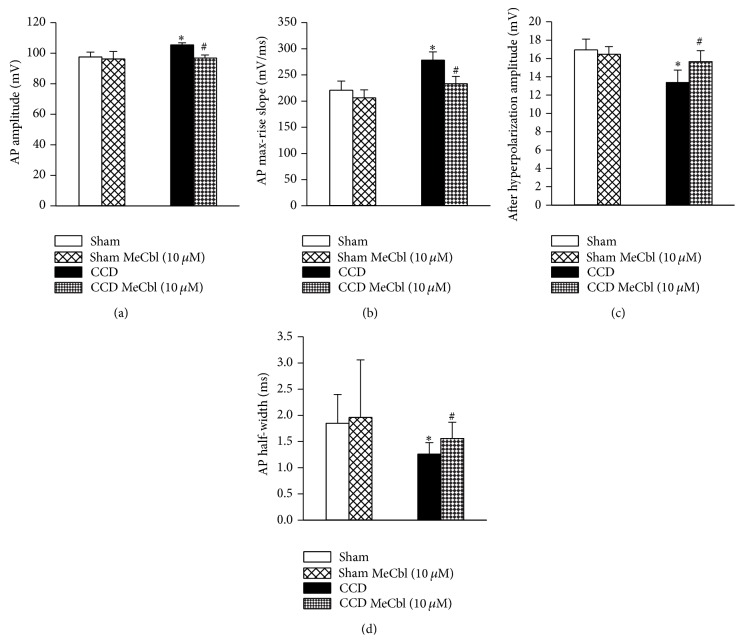
Effects of MeCbl on active membrane properties of medium-sized DRG neurons. Effects of MeCbl on active membrane properties of medium-sized DRG neurons. (a) AP amplitude; (b) AP max-rise slope; (c) AHP amplitude; (d) AP half-width. ^#^ *P* < 0.05, significant differences in active membrane properties of medium-sized DRG neurons compared to those in the absence of MeCbl group; ^∗^ *P* < 0.05, compared with those of sham group.

Compared to sham rats, AP amplitude and max-rise slope were both markedly increased (*Ps* < 0.05, *n* = 32; one-way ANOVA, Figures 4(a) and 4(b)); AP half-width and AHP amplitude were obviously decreased (*Ps* < 0.05, one-way ANOVA, *n* = 32, Figures 4(c) and 4(d)). The increases of AP amplitude and AP max-rise slope and the decreases of AP half-width and AHP amplitude of medium-sized DRG neurons were reversed in the presence of MeCbl in CCD rats (*Ps* < 0.001, Student's *t*-test, *n* = 17, Figures 4(a)–4(d)). Nevertheless, MeCbl did not influence active membrane properties including AP amplitude, max-rise slope, and AP half-width and AHP amplitude of medium-sized DRG neurons in sham rats (*Ps* > 0.05, *n* = 15, Student's *t*-test, Figure 4).

**Figure 5:**
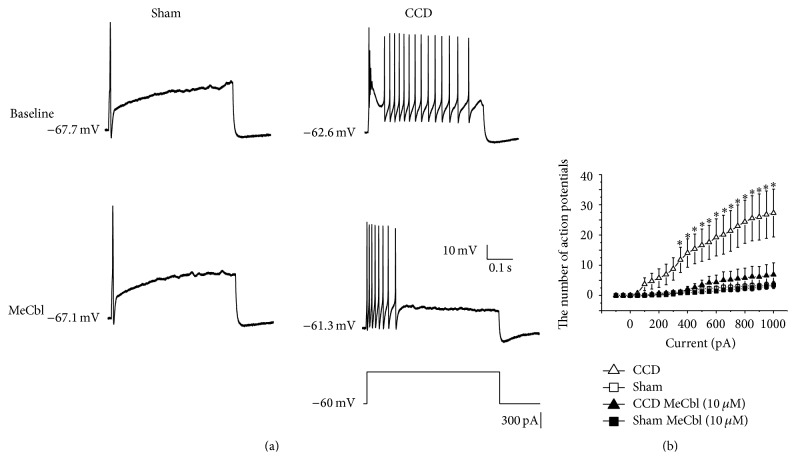
MeCbl inhibited the number of APs of medium-sized DRG neurons. (a) Original traces of APs induced by the depolarized square wave in absence and the presence of MeCbl. (b) Inhibitory effect of MeCbl on the number of action potentials of medium-sized DRG neurons. ^∗^ *P* < 0.05, compared to those in the absence of MeCbl group.

The number of APs was significantly increased in the CCD group than that of sham group (*Ps* < 0.05, one-way ANOVA, Figures 5(a) and 5(b)). The increased number of APs was remarkably decreased by the administration of MeCbl in CCD group (*Ps* < 0.05, repeated-measures ANOVA, Figures 5(a) and 5(b)), but not in the sham group (*Ps* > 0.05, repeated-measures ANOVA, Figures 5(a) and 5(b)).

#### 3.3.2. MeCbl Inhibited the Increase of *I*_*h*_ Current Density in Medium-Sized DRG Neurons

**Figure 6:**
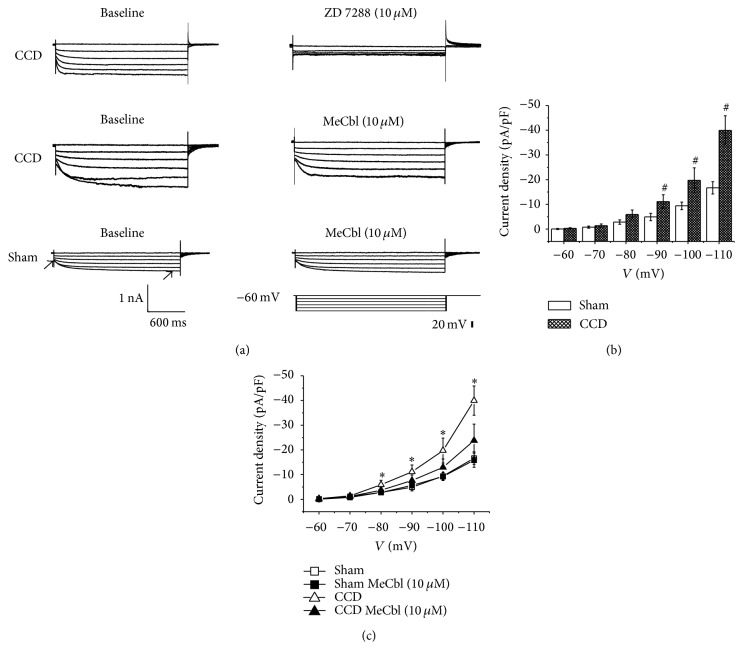
MeCbl inhibited the *I*_*h*_ of medium-sized neurons of DRG neurons. (a) *I*_*h*_ current is evoked by hyperpolarizing voltage steps from a holding potential of −60 mV. *I*_*h*_ current amplitude is calculated from the difference between the steady-state and the instantaneous current. (b) Increased *I*_*h*_ current density of medium-sized DRG neurons in CCD rats. ^#^ *P* < 0.05, compared with those of sham group. (c) Effects of MeCbl on the *I*_*h*_ current density of medium-sized DRG neurons. ^∗^ *P* < 0.05, compared with those in the absence of MeCbl.

About 65% DRG A-type neurons of CCD and sham groups expressed *I*_*h*_ current. *I*_*h*_ current was abolished by ZD7288 (10 *μ*M) but could not be reversed. And it could be absolutely abolished by 2 mM Cs^2+^ (*n* = 4, data not shown) in 5 min and was recovered in 20 min. In contrast to sham group, *I*_*h*_ current densities of medium-sized DRG neurons were increased markedly in CCD rats (*P* < 0.05, one-way ANOVA, Figures 6(a) and 6(b)).

*I*_*h*_ current of medium-sized DRG neurons was recorded after the application of MeCbl* in vitro* (Figure 6(b)). *I*_*h*_ current density was significantly decreased by the perfusion of MeCbl in CCD rats (*n* = 7, *P* < 0.05, Student's *t*-test, Figure 6(c)). However, *I*_*h*_ current density was not affected by the perfusion of MeCbl in sham rats (*n* = 7, *P* < 0.05, Student's *t*-test, Figure 6(c)).

## 4. Discussion

In the present study, we demonstrated that MeCbl inhibited mechanical allodynia in the rat CCD model after a long-term administration and a higher concentration of a short-term treatment of MeCbl. Moreover, MeCbl inhibited the SA, the excitability, and *I*_*h*_ of medium-sized DRG neurons following the CCD. It is likely that MeCbl may exhibit analgesic effect through inhibiting *I*_*h*_ and hyperexcitability of injured DRG neurons.

### 4.1. Continuous Treatment of MeCbl Ameliorated Mechanical Allodynia

MeCbl participates in nervous system maintenance through transmethylating action. Previous studies displayed that MeCbl has widely been used in the treatment of nervous system disorders including diabetic peripheral neuropathy due to promoting myelination and nerve regeneration [35]. Continuous treatment with ultrahigh dose of MeCbl promoted nerve regeneration and myelination [36, 37]. In the present study, we used CCD model, which mimics low back pain and lumbar spinal stenosis syndromes well, to evaluate the effect of monotherapy of MeCbl. Continuous administration of a dose of MeCbl (2.5 mg/kg/day) higher than that used in previous reports (500 *μ*g–1 mg/kg/day) was used. Our results showed that a continuous treatment of high dose MeCbl markedly alleviated tactile allodynia in the CCD rat, while low and medium dose MeCbl could not. Moreover, a short-term administration of high dose MeCbl also exhibited an analgesic effect on mechanical allodynia. Thus, our results here confirmed similar results that continuous administration of high dose MeCbl could ameliorate neuropathic pain associated with diabetic neuropathy [12, 13] and also showed that the single treatment of an extra high dose of MeCbl ameliorated mechanical allodynia. But our results were not consistent with previous report that vitamin B12 or the coapplication of MeCbl and vitamin E did not alter mechanical allodynia in sciatic nerve crush injured rats [11, 38]. The discrepancy is very likely due to different doses and periods of MeCbl treatment as only high dose and long-term treatment of MeCbl may be effective in treating mechanical allodynic behavior. It is proposed that mechanical hyperalgesia may involve different mechanisms from those of the thermal hyperalgesia. Further studies are necessary to address the different efficacy of MeCbl on thermal hyperalgesia and mechanical allodynia.

### 4.2. MeCbl Inhibited the SA and the Excitability of A-Type DRG Neurons

MeCbl acts as a coenzyme of methionine synthase, which transforms homocysteine to methionine in the methylation cycle including methylation of DNA or proteins [5]. Although several studies showed that MeCbl improved nerve regeneration and nerve conduction velocity via altering activation of cellular signaling pathway in peripheral neuropathy models, precise analgesic mechanism of MeCbl is still needed to be clarified. For example, continuous administration of high doses of MeCbl not only facilitated neurite outgrowth and neuronal survival but also improves nerve regeneration and functional recovery through activation of protein kinases extracellular regulated protein kinases 1/2 (Erk1/2) and protein kinase B (Akt) in sciatic nerve injury model [6]. MeCbl upregulated the expression of neural insulin-like growth factor-1 (IGF-1) gene [39]. MeCbl normalized altered PKC activities in experimental diabetic neuropathy [40]. However, the downstream target of signaling pathway of MeCbl remains obscure.

Previous studies showed that DRG neurons exhibited the hyperexcitability in the CCD rat, such as SA, increased excitability, and upregulated *I*_*h*_ [22–25]. Our study showed that continuous treatment of MeCbl exerted an antiallodynic behavior in the CCD rat. The low-threshold type of medium-sized DRG neurons may become sensitized after tissue inflammation or peripheral nerve injury [41–43]. Thus, the effect of MeCbl was further determined on the SA and the excitability of medium-sized DRG neurons. Our results provided the experimental evidence that MeCbl significantly inhibited the SA of A-type dorsal root fibers in a dose-dependent way* in vivo* and the SA from medium-sized DRG neurons* in vitro* from the CCD rat. It indicated that the analgesic mechanism of MeCbl improved mechanical allodynia through inhibiting the SA of medium-sized DRG neurons and A-type dorsal root fibers in neuropathic pain state.

Recently, several studies showed that medium-sized DRG neurons exhibited hyperexcitability, such as an increased sodium current, a decreased delayed rectifier voltage-dependent potassium current, and a decreased threshold for action potential [25, 44]. According to conduction velocity, medium-sized DRG neurons recorded were considered as A*δ* DRG neurons in the present study [45]. Our results showed that the amplitude and the max-rise slope of AP were significantly increased, and AP half-width and AHP amplitude were markedly decreased in medium-sized DRG neurons of CCD rats. It is consistent with LaMotte's reports that excitability of compressed medium-sized DRG neurons might be obviously increased [25, 44]. Increased max-rise slope of AP and AP amplitude and decreased AP half-width may be due to an increased sodium current in medium-sized DRG neurons following the compression of the DRG. It is reported that AHP amplitude was augmented by *I*_*h*_ inhibitor ZD7288 [46]. Our results provide new evidence that AHP amplitude was decreased in medium-sized DRG neurons, indicating an increased *I*_*h*_ in those neurons of CCD rats.

Our results showed that MeCbl significantly decreased these increased characters of AP amplitude and the max-rise slope in CCD rats, and increased the decrease of AP half-width and AHP amplitude as well. And MeCbl obviously suppressed the number of APs induced after nerve injury. It was suggested that MeCbl inhibited the increased excitability of medium-sized DRG neurons in CCD rats. The half-life of vitamin B12 in serum ranges from approximately 20 to 50 min, and one administration may not maintain a high concentration of vitamin B12 [47]. Therefore, to maintain the serum concentration of vitamin B12 to some extent, we continuously administered a higher dose. Our results showed an analgesic effect on mechanical allodynia following a single injection of an extra high dose MeCbl, while continuous treatment of minimal MeCbl may overcome the fast degeneration and maintain a high serum concentration of MeCbl. Therefore, continuous treatment of a lower dose of MeCbl showed an obvious and a stable analgesic efficiency. Combined with behavioral data, our results suggested that continuous treatment of high dose MeCbl might exhibit an antiallodynia via inhibiting the hyperexcitability of medium-sized DRG neurons in neuropathic pain state.

### 4.3. MeCbl Inhibited *I*_*h*_ in Medium-Sized DRG Neurons

*I*_*h*_ has been implicated in nociception and chronic pain. The ion channels underlying *I*_*h*_ are the hyperpolarization-activated cyclic nucleotide-gated (HCN) family [48]. HCN channels are activated by membrane hyperpolarization, are permeable to sodium and potassium ions, and comprise four isoforms, HCN1–HCN4. HCN1 and HCN2 contribute to different amplitude and properties of *I*_*h*_ in nociceptive or low-threshold mechanoreceptive sensory neurons [49, 50]. Previous studies showed that *I*_*h*_ was upregulated in medium-sized DRG neurons [24, 29]. More recent studies showed increased HCN2 subunit mediated mechanical allodynia after peripheral nerve injury and inflammation [51, 52]. Our results showed that the magnitude of *I*_*h*_ was increased significantly in medium-sized DRG of CCD rats, consistent with other studies. Furthermore our results showed that MeCbl significantly reversed the decrease of AHP amplitude and the increase of *I*_*h*_ in medium-sized DRG neurons. It is suggested that MeCbl may inhibit *I*_*h*_ and then suppress the SA and the hyperexcitability of medium-sized DRG neurons in neuropathic pain state. MeCbl may exhibit antiallodynia through inhibiting *I*_*h*_ and may further inhibit the SA and the hyperexcitability of injured medium-sized DRG neurons. MeCbl participates in nervous system maintenance through the methylation of DNA or proteins. It is very likely that MeCbl inhibited the *I*_*h*_, the hyperexcitability of medium-sized DRG neurons in an epigenetic modulation. Therefore, the mechanism underlying the inhibition of MeCbl on *I*_*h*_ and the hyperexcitability needs to be determined in further study.

MeCbl is regarded as a potential vitamin of neuropathic pain killer with excellent tolerance and safety at the moment [35]. Continuous treatment with high dose of MeCbl ameliorates mechanical allodynia via the inhibition of SA of A-type DRG neurons, the excitability, and *I*_*h*_ current density of medium-sized DRG neurons in the animal model of low back pain. Thus, high dose MeCbl has great potential for treating peripheral neuropathy. Further investigations with MeCbl may help elucidate its mechanisms of action, which may further enable us to treat peripheral neuropathic pain.

## Figures and Tables

**Table 1:** The effect of MeCbl (10 *μ*M) on passive membrane properties of medium-sized DRG neurons.

	*n*	Membrane potential (mV)	Cm (pF)	Rm (MΩ)
Sham	15	−69.00 ± 1.36	64.14 ± 10.93	49.37 ± 8.19
Sham + MeCbl	15	−66.20 ± 1.77	65.83 ± 10.68	48.24 ± 8.17
CCD	17	−66.45 ± 1.22	86.00 ± 19.16	62.74 ± 10.91
CCD + MeCbl	17	−64.12 ± 1.86	77.01 ± 8.84	52.32 ± 8.08

No significant differences in membrane potential, Cm (membrane capacitance), and Rm (membrane resistance) in medium-sized DRG neurons of CCD rats in the presence of MeCbl (*Ps* > 0.05, Student's *t*-test).
